# The Impacts of Age and Sex in a Mouse Model of Childhood Narcolepsy

**DOI:** 10.3389/fnins.2021.644757

**Published:** 2021-03-04

**Authors:** Alissa A. Coffey, Adam A. Joyal, Akihiro Yamanaka, Thomas E. Scammell

**Affiliations:** ^1^Department of Neurology, Beth Israel Deaconess Medical Center and Harvard Medical School, Boston, MA, United States; ^2^Department of Neuroscience II, Research Institute of Environmental Medicine, Nagoya University, Nagoya, Japan

**Keywords:** orexin, narcolepsy, cataplexy, age, sex, mice, pediatric, childhood

## Abstract

Narcolepsy is a sleep disorder caused by selective death of the orexin neurons that often begins in childhood. Orexin neuron loss disinhibits REM sleep during the active period and produces cataplexy, episodes of paralysis during wakefulness. Cataplexy is often worse when narcolepsy develops in children compared to adults, but the reason for this difference remains unknown. We used *orexin-tTA; TetO DTA* mice to model narcolepsy at different ages. When doxycycline is removed from the diet, the orexin neurons of these mice express diphtheria toxin A and die within 2–3 weeks. We removed doxycycline at 4 weeks (young-onset) or 14 weeks (adult-onset) of age in male and female mice. We implanted electroencephalography (EEG) and electromyography (EMG) electrodes for sleep recordings two weeks later and then recorded EEG/EMG/video for 24 h at 3 and 13 weeks after removal of doxycycline. Age-matched controls had access to doxycycline diet for the entire experiment. Three weeks after doxycycline removal, both young-onset and adult-onset mice developed severe cataplexy and the sleep-wake fragmentation characteristic of narcolepsy. Cataplexy and maintenance of wake were no worse in young-onset compared to adult-onset mice, but female mice had more bouts of cataplexy than males. Orexin neuron loss was similarly rapid in both young- and adult-onset mice. As age of orexin neuron loss does not impact the severity of narcolepsy symptoms in mice, the worse symptoms in children with narcolepsy may be due to more rapid orexin neuron loss than in adults.

## Introduction

Orexins are wake-promoting neuropeptides necessary for the maintenance of long periods of wakefulness and the regulation of REM sleep (Saper et al., 2001; Lu et al., 2006; Branch et al., 2016; Chowdhury et al., 2019). Narcolepsy is caused by severe loss of the orexin-producing neurons in the hypothalamus, and the resulting symptoms of narcolepsy include excessive daytime sleepiness, the occurrence of REM sleep at any time of day, and cataplexy – episodes of muscle atonia during wakefulness that are likely produced by some of the same neural mechanisms that produce atonia during REM sleep (Mahoney et al., 2019). Most patients develop narcolepsy before the age of 25, most commonly between age 10 and 20 (Yoss and Daly, 1960; Dauvilliers et al., 2001; Ohayon et al., 2005; Longstreth et al., 2009).

The symptoms of narcolepsy are usually more severe when the disease begins in childhood compared to adults. Children with narcolepsy are often sleepier than adults with narcolepsy, as indicated by shorter sleep latencies on the Multiple Sleep Latency Test (Young et al., 1988) and more total sleep over 24 h (Pizza et al., 2013). In adults, cataplexy is usually triggered by strong, positive emotions, but children can have spontaneous cataplexy (Serra et al., 2008; Overeem et al., 2011; Plazzi et al., 2011). Cataplexy typically lasts only 1–2 min in adults (Overeem et al., 2011), but children with narcolepsy can have status cataplecticus, periods of muscle weakness lasting hours which is extremely rare in adults (Quinto et al., 2005; Simon et al., 2004; Calabro et al., 2007; Ping et al., 2007; Panda, 2014; Antelmi et al., 2017). Nearly half of young patients report that cataplexy is their most disruptive symptom, yet even with treatment, more than 40% have cataplexy every day (Maski et al., 2017). Though longitudinal studies are sparse, it appears that this severe sleepiness and cataplexy with childhood-onset narcolepsy lessens over a few years, developing into the pattern typical of adults (Plazzi et al., 2011; Pizza et al., 2013). Narcolepsy onset in younger children is particularly disruptive because it is associated not only with more severe symptoms but also with precocious puberty and obesity, indicating multisystem disruption (Kotagal et al., 1990, 2004; Plazzi et al., 2006; Vendrame et al., 2008; Poli et al., 2013; Ponziani et al., 2016).

It is currently unknown why symptoms of narcolepsy are so severe with disease onset in childhood. To address this question, we compared the effects of orexin neuron loss in young mice vs. adult mice using *orexin-tTA; TetO DTA* mice, a novel mouse model that enables control over of the timing of orexin neuron loss (Tabuchi et al., 2014).

## Materials and Methods

### Animals

All experiments were approved by the Institutional Animal Care and Use Committee of Beth Israel Deaconess Medical Center and Harvard Medical School and were performed in accordance with the National Institutes of Health *Guide for the Care and Use of Laboratory Animals*.

To induce orexin neuron death, we used male and female *orexin-tTA; TetO DTA* mice (Tabuchi et al., 2014). These mice express diphtheria toxin A (DTA) specifically in the orexin neurons under a Tet-off system. When mice have access to chow containing doxycycline (DOX), the orexin neurons are healthy and sleep/wake behavior is normal. However, after DOX removal, the orexin neurons express DTA and die within 2–3 weeks. Cataplexy begins around 3 weeks after DOX removal and increases until it plateaus 8–10 weeks later (Tabuchi et al., 2014).

All animals were housed in a temperature-controlled (22 ± 1.4°C) vivarium on a 12:12 h light:dark cycle with regular mouse chow or DOX chow (100 mg/kg by weight, Envigo) and water available *ad libitum*. DOX chow was stored at 4°C and changed weekly (as per vendor instructions) to prevent degradation of the DOX at room temperature. Genotyping was performed using real-time PCR (Transnetyx).

### Orexin Neuron Loss

**FIGURE 1:**
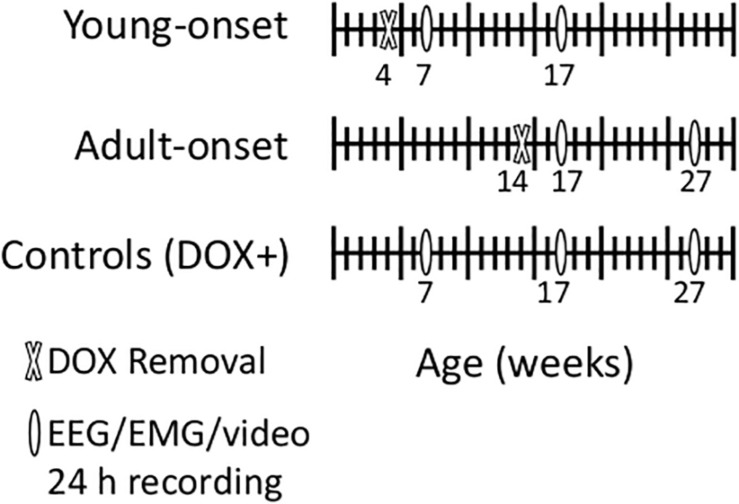
Schematic of experimental design. We removed doxycycline (DOX) from the chow of young-onset mice at 4 weeks of age and of adult-onset mice at 14 weeks of age. We recorded EEG, EMG and video for characterization of sleep/wake/cataplexy 3 and 13 weeks later. We also recorded from age-matched control mice that were maintained on doxycycline the entire experiment (DOX+).

Mating pairs of *orexin-tTA; TetO DTA* mice were fed DOX chow so that litters would receive DOX *in utero* via maternal circulation and postnatally via lactation. We removed DOX from the diet (DOX−) of *orexin-tTA; TetO DTA* mice at age 4 weeks (young-onset group) or 14 weeks (adult-onset group) (Figure 1). These ages were chosen because a 4-week-old mouse is about the same developmental age as an 11–12 years old child, and a 14-week old mouse is roughly equivalent to an early-20s human (Flurkey et al., 2007).

### Study Design

We implanted mice with EEG/EMG electrodes at 6 or 16 weeks of age to record cataplexy and sleep/wake behavior. We recorded brain activity using electroencephalography (EEG), muscle activity using electromyography (EMG), and general behavior using infrared video to aid in identification of sleep/wake behavior and cataplexy detection.

In adult *orexin-tTA; TetO DTA* mice, cataplexy begins about 3 weeks after DOX removal and plateaus 11–13 weeks after DOX removal (Tabuchi et al., 2014). Therefore, for young-onset mice (DOX removed at 4 weeks of age), we recorded sleep/wake behavior at 7 and 17 weeks of age. For adult-onset mice (DOX removal at 14 weeks of age), we recorded behavior at 17 and 27 weeks of age to control for time since DOX removal. In addition, we recorded from age-matched *orexin-tTA; TetO DTA* controls maintained on DOX chow for the entire experiment (DOX+ with recordings at age 7, 17, and 27 weeks) to control for any age-related changes in sleep/wake behavior. Each group contained male and female mice. Some longitudinal recordings were disrupted by the 2020 COVID-19 lab shutdown, so this experiment includes both longitudinal (repeated recordings in the same mouse) and cross-sectional (only one recording from a mouse) data. The total numbers of mice were: DOX+ 7 weeks (seven males, seven females); DOX+ 17 weeks (seven males, including three longitudinal recordings; nine females, including four longitudinal recordings); DOX+ 27 weeks (five males, including four longitudinal recordings; 10 females including seven longitudinal recordings); young-onset 7 weeks (seven males, seven females); young-onset 17 weeks (eight males, including four longitudinal recordings; six females including two longitudinal recordings); adult-onset 17 weeks (eight males, six females); adult-onset 27 weeks (10 males, including five longitudinal recordings; nine females including three longitudinal recordings).

### Surgery

We anesthetized mice using ketamine/xylazine (100/10 mg/kg, i.p.) and placed them into a stereotaxic frame. We soldered leads made from multistranded stainless steel wire (Cooner Wire, part number AS633) to two stainless steel screws which were implanted into the skull (1 mm lateral and 1 mm rostral to bregma, 1 mm lateral to bregma, and 1 mm rostral to lambda). We implanted two EMG electrodes made from Cooner wire into the neck extensor muscles. All leads were soldered to a 2 × 2 pin microstrip connector which we secured to the skull using dental cement. We treated each mouse with Meloxicam SR (4 mg/kg, s.c.) immediately after surgery.

### EEG/EMG/Video Recordings and Analysis

After at least 1 week of recovery, we moved mice into the recording chambers to allow at least 5 days of acclimation to the recording cage and EEG/EMG cable. During recordings, EEG/EMG signals were amplified, filtered (EEG: 0.3–35 Hz; EMG: 100–300 Hz; Grass Amplifier 6SS, Grass Instruments), and digitized at a sampling rate of 256 Hz (VitalRecorder, Kissei Comtec) with simultaneous infrared video recordings. We scored sleep/wake signals in 10 s epochs semiautomatically using SleepSign (Kissei Comtec, band pass filter settings: EEG, 0.25–64 Hz; EMG, 10–60 Hz, with a notch filter at 60 Hz for each) and performed manual corrections as needed. We scored cataplexy manually using EEG/EMG and video according to a consensus definition (Scammell et al., 2009). Specifically, we scored an event as cataplexy if four criteria were met: the episode was (1) an event of nuchal atonia lasting at least 10 s, (2) the mouse was not asleep during the 40 s preceding the episode, (3) the mouse was immobile for the duration of the event, and (4) the EEG was dominated by theta activity (Scammell et al., 2009).

### Confirmation of Orexin Neuron Loss

After the recordings, we perfused all mice and immunostained brains as outlined below to confirm orexin neuron loss in DOX− mice and to confirm that the genetic construct of the *orexin-tTA; TetO DTA* mice did not cause any unexpected orexin neuron loss in the DOX + control mice. In addition, we immunostained brains from young-onset and adult-onset mice perfused 0, 1, 2, and 3 weeks after DOX removal (*n* = 6–11 mice per group, including males and females) to test whether any differences in cataplexy could be due to different rates of orexin neuron loss.

### Immunohistochemistry and Neuron Counting

We anesthetized mice with ketamine/xylazine (150/15 mg/kg i.p.) and transcardially perfused them with 30 mL phosphate-buffered saline (PBS, pH = 7.4) and 30 mL of 10% buffered formalin (pH = 7). We then harvested brains and post-fixed them in 10% formalin for 24–48 h. After fixation, we transferred brains to a 30% sucrose solution in PBS-azide for 48–72 h.

The orexin field spans the lateral and posterior hypothalamus (De Lecea et al., 1998; Sakurai et al., 1998), so we collected 30 μm sections in a 1:3 series from bregma −0.94 mm to −2.80 mm to capture the full orexin field across ∼20 sections in each series.

We immunostained one series for orexin-A to compare the number of orexin neurons at different time points across groups. We first rinsed sections three times with PBS for 5 min before incubating them for 30 min in 0.3% hydrogen peroxide in PBS with Triton (0.25% Triton X-100 in PBS) to quench endogenous peroxidases. Next, we rinsed sections again in PBS three times for 5 min before blocking them with 3% normal horse serum (NHS) for 2 h. We followed blocking with a primary overnight incubation in goat anti-orexin-A antibody (1:5,000; Santa Cruz SC-8070, Lot: C0512) in 0.02% sodium azide in PBT and 3% NHS.

The next day, we began by rinsing sections six times for 5 min in PBS and then incubated them in biotinylated donkey anti-goat IgG secondary antiserum (1:500; Jackson ImmunoResearch 705-065-147, lots: 129472, 150417) in 3% NHS in PBT for 2 h. After secondary incubation, we again rinsed sections three times for 10 min in PBS followed by a 1 h incubation in avidin-biotin complex in PBS (Vector Laboratories PK-6100, lot: ZF1218). To stain orexin-A brown, we then placed sections in 3,3′-diaminobenzidine (DAB) (Vector SK-4100, Lot: SLCD1660) in tris-buffered saline (TBS) and 0.024% hydrogen peroxide for 6 min. After the reaction, we again rinsed sections three times for 5 min in PBS before mounting them on Superfrost Plus slides and letting them dry overnight. All incubations and washes were carried out at room temperature on a shaker. We dehydrated the sections using graded ethanol for 3 min per step, followed by clearing with xylenes, and then coverslipped the slides using Cytoseal 68 mounting media (Thermo Fisher Scientific, 23-244256).

We imaged sections using bright field microscopy under the 5× lens of an Axioplan2 microscope (Zeiss) and captured images using AxioCam HRC (Zeiss). Finally, we analyzed the images and counted orexin-A immunoreactive neurons using ImageJ’s Multi-Point tool. All immunostaining and cell counting were performed by AJ for consistency.

### Statistical Analysis

We performed statistical analysis in R version 4.0.2 using the following packages: nlme, sjPlot, sjmisc, ggplot2, and plyr (Wickham, 2011, 2016; Lüdecke, 2018, 2020; Pinheiro et al., 2020; R Core Team, 2020). We performed separate linear mixed multilevel regressions for each dependent variable of interest: percent time in cataplexy (during the dark period, light period, and over 24 h), number of cataplexy bouts (during the dark period, light period, and over 24 h), duration of cataplexy bouts (during the dark period), percent time awake (during the dark period, light period, and over 24 h), number of wake bouts (during the dark period), duration of wake bouts (during the dark period), percent time in NREM sleep (during the dark period, light period, and over 24 h), and percent time in REM sleep (during the dark period, light period, and over 24 h). The DOX+ control group was excluded from the cataplexy regressions because no control mice had cataplexy and including the group would violate the homoscedasticity assumption of the regression. Independent variables for the cataplexy regressions included: group (young-onset and adult-onset), sex (female and male), time since DOX removal, and all interaction terms (group × sex, sex × time, group × time, group × sex × time). Independent variables for the sleep/wake regressions included: group (young-onset, adult-onset, and DOX+), sex (female and male), age, and all interaction terms (group × sex, sex × age, group × age, group × sex × age). There were two potential sources of correlation in our data: mice within a litter and observations within a mouse (we recorded from some mice multiple times). To account for these, we included a litter-specific random intercept and an observation-specific random intercept in each model. We assumed a normal distribution and an unknown standard error for these random intercepts and the residual error. We log-transformed (base 10) bout durations of wake and cataplexy for each mouse to prevent violation of the normality assumption of the regressions.

For the orexin neuron loss confirmation experiment, we ran a 2 × 2 × 4 analysis of variance (ANOVA) including orexin neuron count as the dependent variable and group (young-onset and adult-onset), sex (male and female), time since DOX removal (0, 1, 2, 3 week), and the relevant interaction terms (group × time, sex × time, group × sex × time) as the independent variables.

## Results

### Age of Orexin Neuron Loss Did Not Impact Cataplexy

**FIGURE 2:**
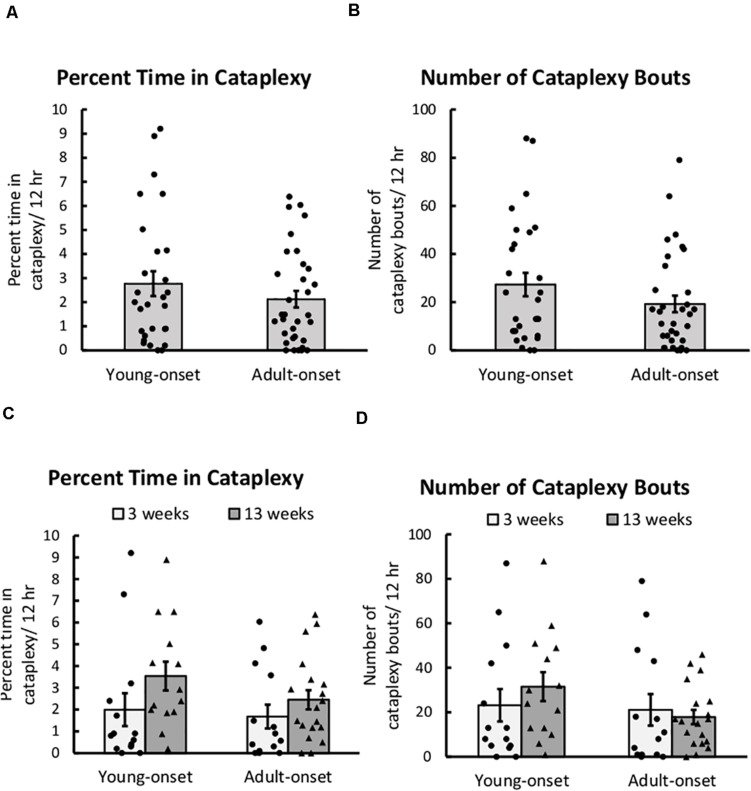
Cataplexy was unaffected by age of orexin neuron loss. **(A)** Average percent of time young-onset and adult-onset mice spent in cataplexy during the 12 h dark period, collapsed across age. **(B)** Average number of cataplexy bouts during the 12 h dark period in young-onset and adult-onset mice, collapsed across age. **(C)** Average percent time spent in cataplexy bouts during the dark period in young-onset and adult-onset mice at 3 or 13 weeks after doxycycline removal. **(D)** Average number of cataplexy bouts during the dark period in young-onset and adult-onset mice at 3 or 13 weeks after doxycycline removal. All data are presented as mean ± SEM with individual data points overlaid.

Young-onset and adult-onset mice had similar amounts of severe cataplexy. The main effect of group (young-onset vs. adult-onset) was not significant for either the percentage of time spent in cataplexy or the number of cataplexy bouts during the dark period (Figure 2). The group × sex, group × time since DOX removal, and group × sex × time since DOX removal interactions were all non-significant for the percentage of time spent in cataplexy and for the number of cataplexy bouts. Still, the number of bouts tended to increase over time since DOX removal in young-onset mice but not in adult-onset mice, *t*(10) = −1.89, *p* = 0.087 (Figure 2D). Cataplexy mainly occurs in the dark period, so we present all analyses on cataplexy during the dark period. Cataplexy was much less common in the light period, and results were similar for cataplexy during the light period or across the 24 h period. As cataplexy occurs only during wakefulness, cataplexy amounts could differ due to differences in time spent awake. For this reason, we also analyzed the amount of cataplexy as a percent of time awake and the number of cataplexy bouts per hour of wakefulness in all mice, but the conclusions were the same, indicating that differences in cataplexy were unrelated to differences in wake time were unrelated to differences in wake time. For representative EEG/EMG traces and video during a cataplexy bout in example young-onset and adult-onset mice, see Supplementary Videos.

**FIGURE 3:**
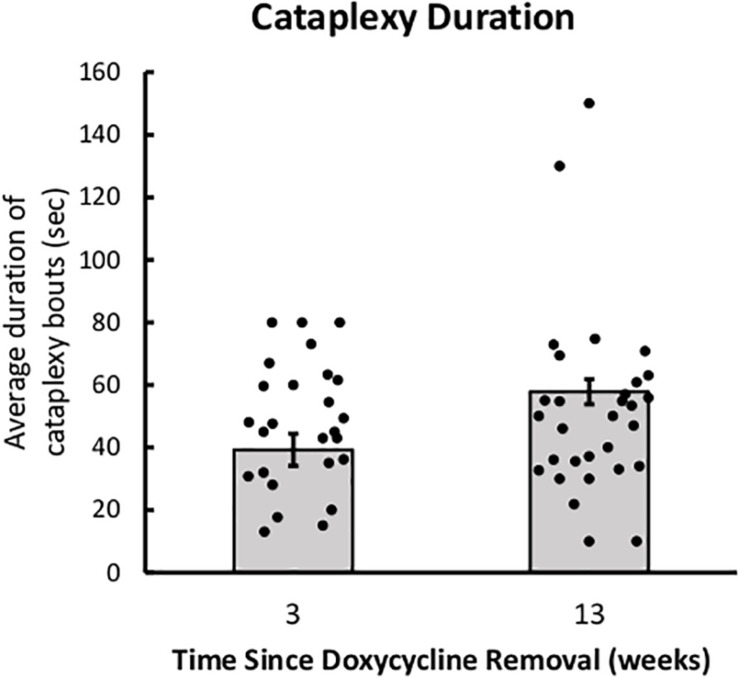
Cataplexy duration increases across time since DOX removal. Average duration of cataplexy bouts during the dark period in both young-onset and adult-onset mice at 3 and 13 weeks after doxycycline removal.

There were no group differences in the duration of cataplexy bouts, but bouts of cataplexy tended to lengthen over time since DOX removal, *t*(8) = 2.01, *p* = 0.079 (Figure 3). On average, for every day since DOX removal, cataplexy bouts were about 1 s longer.

### Female Mice Had More Cataplexy Than Male Mice

**FIGURE 4:**
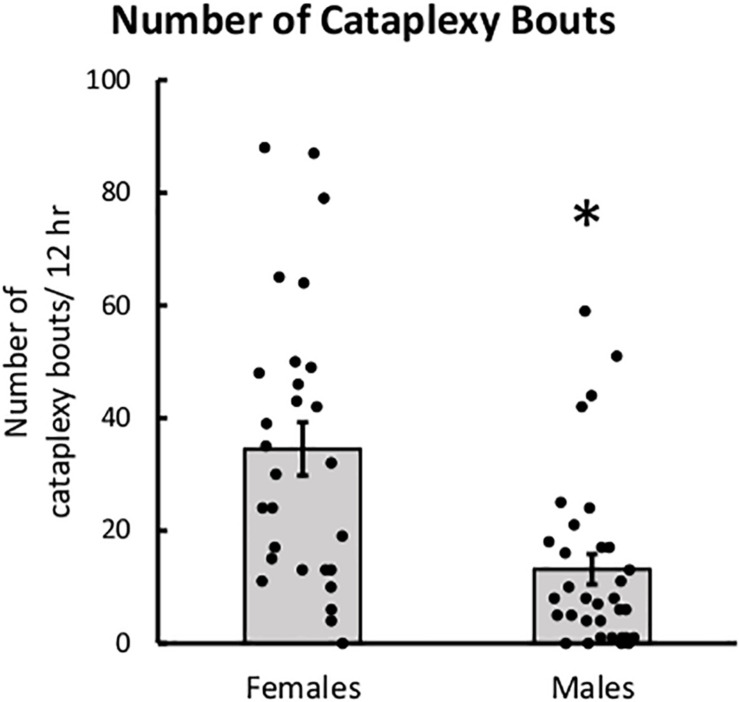
Female mice had more cataplexy than males. Average number of cataplexy bouts during the 12-h dark period in female and male mice, collapsed across age. ^∗^*p* < 0.05.

Female mice had more bouts of cataplexy during the dark period than male mice. The main effect of sex was significant for the number of cataplexy bouts in the dark period, *t*(21) = −2.20, *p* = 0.039, indicating that male mice have fewer bouts of cataplexy than female mice (Figure 4). The main effect of sex was not significant for percent of time spent in cataplexy. No interaction effects were significant for either percent time or number of cataplexy bouts during the dark period.

Female mice also spent more time in cataplexy during the light period and across the 24 h period than male mice. The main effect of sex was significant for the percentage of time spent in cataplexy during the light period, *t*(21) = −2.85, *p* = 0.0095, and across the 24 h period, *t*(21) = −2.23, *p* = 0.037. The main effect of sex was also significant for the number of cataplexy bouts during the light period, *t*(21) = −2.63, *p* = 0.016, and across the 24 h period, *t*(21) = −2.59, *p* = 0.017. Interestingly, the age × sex interaction for percent time spent in cataplexy during the light period was nearly significant, *t*(10) = 2.199, *p* = 0.052. While female mice spent more time in cataplexy during the light period at 3 weeks after DOX removal, male and female mice had similar amounts of cataplexy by 13 weeks after DOX removal. The same trend was seen in the number of cataplexy bouts during the light period, age × sex interaction, *t*(10) = 1.96, *p* = 0.078.

### Age of Orexin Neuron Loss Did Not Impact REM Sleep

**FIGURE 5:**
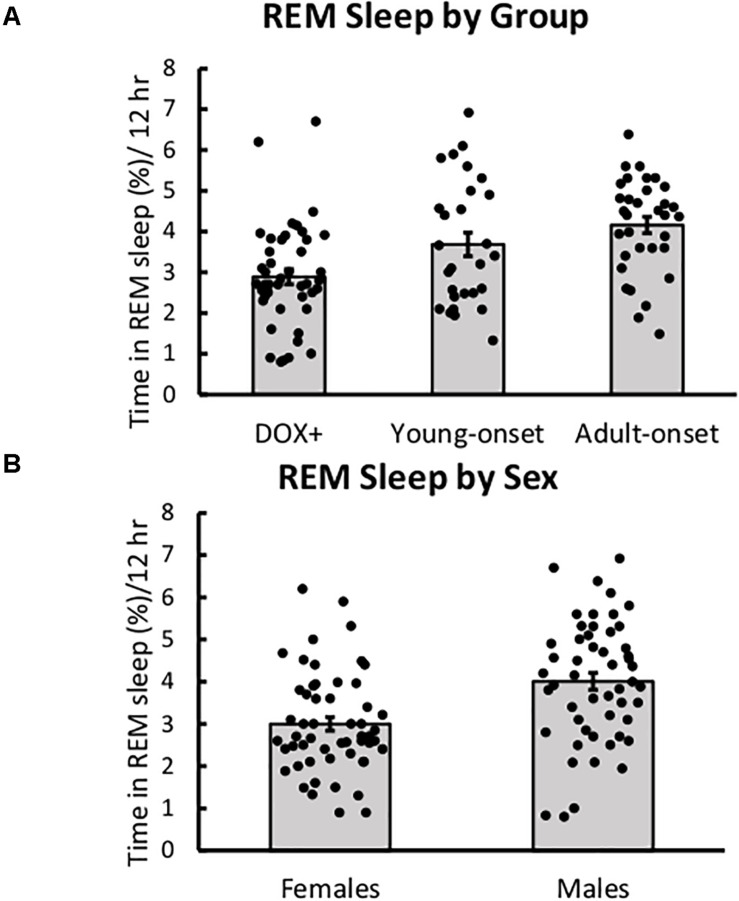
REM sleep was unaffected by age of orexin neuron loss. **(A)** Average percent time spent in REM sleep during the 12-h dark period in DOX+ controls, young-onset mice, and adult-onset mice, collapsed across age. **(B)** Average percent time spent in REM sleep during the 12-h dark period in female and male mice.

There were no significant main or interaction effects on the percentage of time spent in REM sleep during the dark period (Figure 5). Still, young-onset mice tended to spend more time in REM sleep during the dark period than DOX+ controls, *t*(43) = 1.86, *p* = 0.07, and male mice tended to spend more time in REM sleep than females, *t*(43) = 1.94, *p* = 0.059.

Young-onset female mice spent less time in REM during the light period than DOX+ female mice, but DOX+ and young-onset males spent similar amounts of time in REM. Group × sex interaction, *t*(42) = 2.25, *p* = 0.029.

### Orexin Neuron Loss Resulted in Poor Maintenance of Wake

**FIGURE 6:**
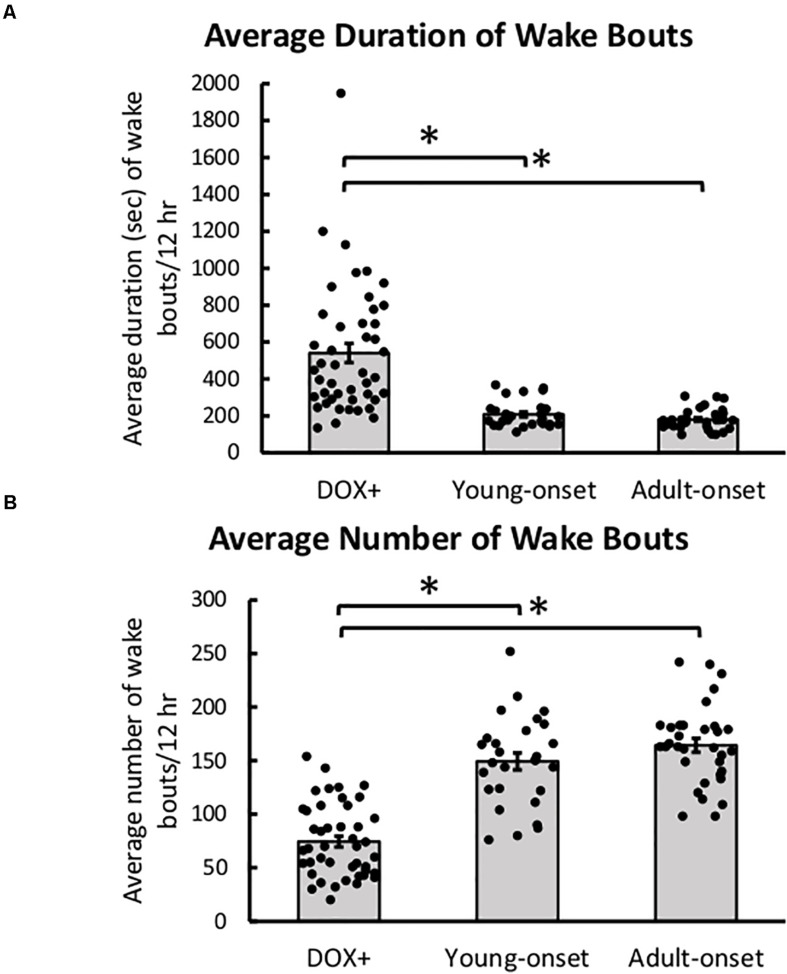
Poor maintenance of wake with orexin neuron loss. **(A)** Average duration of wake bouts and **(B)** average number of wake bouts in DOX+ (control), young-onset, and adult-onset mice. *p < 0.05.

Young-onset and adult-onset mice had significantly shorter wake bouts during the dark period than DOX+ controls, *t*(43) = −3.90, *p* = 0.0003 and *t*(43) = −2.88, *p* = 0.0062, respectively (Figure 6A). Wake durations were shorter in male DOX+ controls than females but the same between adult-onset males and females so the reduction in wake bout durations was steeper in female adult-onset mice than males [group × sex interaction, *t*(43) = 2.13, *p* = 0.039]. This interaction was not seen in young-onset mice.

Young-onset and adult-onset mice also had more wake bouts during the dark period than DOX+ controls [*t*(43) = 3.61, *p* = 0.0008, and *t*(43) = 3.90, *p* = 0.0003, respectively] (Figure 6B). DOX+ male mice had more bouts of wake than DOX+ female mice, but adult-onset male and female mice had similar numbers of wake bouts so the increase in number of wake bouts was steeper in female adult-onset mice than males [group × sex interaction, *t*(43) = −3.26, *p* = 0.0022].

Young-onset mice spent less time awake during the dark period than DOX+ controls, *t*(43) = −2.53, *p* = 0.015, and tended to spend more time in NREM sleep during the dark period than DOX+ controls, *t*(43) = 1.96, *p* = 0.056, but this effect was not significant (Table 1). Cataplexy in the dark period in young-onset mice likely explains this difference in wake time. No such differences were seen in the adult-onset mice.

**TABLE 1:** Time spent in each sleep/wake state during the dark period and the light period.

	**DOX+ 7 weeks (*n* = 14)**	**Young-onset 7 weeks (*n* = 14)**	**DOX+ 17 weeks (*n* = 16)**	**Young-onset 17 weeks (*n* = 14)**	**Adult-onset 17 weeks (*n* = 14)**	**DOX+27 weeks (*n* = 15)**	**Adult-onset 27 weeks (*n* = 19)**
**Dark Period**							
Wake	74.1 (1.5)	66.0 (1.6)	71.7 (2.2)	68.8 (2.5)	67.4 (2.4)	71.4 (2.7)	63.7 (2.0)
NREM sleep	22.9 (1.4)	27.7 (1.4)	25.4 (2.0)	24.7 (2.4)	26.6 (2.4)	25.8 (2.5)	29.8 (2.0)
REM sleep	3.0 (0.2)	4.3 (0.4)	2.9 (0.4)	3.0 (0.4)	4.3 (0.3)	2.8 (0.4)	4.0 (0.3)
Cataplexy	0 (0)	2.0 (0.8)	0 (0)	3.5 (0.7)	1.7 (0.5)	0 (0)	2.5 (0.4)
**Light period**							
Wake	37.7 (3.1)	39.4 (3.1)	37.5 (1.2)	40.4 (1.1)	42.2 (1.4)	37.1 (1.4)	38.7 (1.2)
NREM sleep	46.6 (3.8)	48.6 (0.9)	54.1 (1.0)	51.9 (1.0)	49.3 (1.2)	53.5 (1.4)	53.8 (1.2)
REM sleep	8.6 (0.8)	8.4 (0.5)	8.4 (0.3)	7.1 (0.3)	8.1 (0.4)	9.3 (0.3)	7.1 (0.3)
Cataplexy	0 (0)	0.4 (0.2)	0 (0)	0.4 (0.1)	0.2 (0.1)	0 (0)	0.4 (0.1)

*Data are presented as mean percent time/12 h (SEM).*

Age of orexin neuron loss did not affect the percentage of time spent awake, in NREM sleep, or in REM sleep across the 24 h period.

Considered together, these results support the perspective that orexin neuron loss results in poor maintenance of wake, a common symptom of orexin deficiency in mouse models and human narcolepsy (Hara et al., 2001; Mochizuki et al., 2004; Tabuchi et al., 2014; Scammell, 2015).

### Age of Orexin Neuron Loss Mildly Affected NREM Sleep

Adult-onset mice spent less time in NREM sleep during the light period than DOX+ controls, *t*(42) = −2.06, *p* = 0.045, but there was no difference in percent time in NREM sleep during the light period between young-onset mice and DOX+ controls (Table 1).

### Orexin Neuron Numbers Declined Similarly in Young and Adult Mice

**FIGURE 7:**
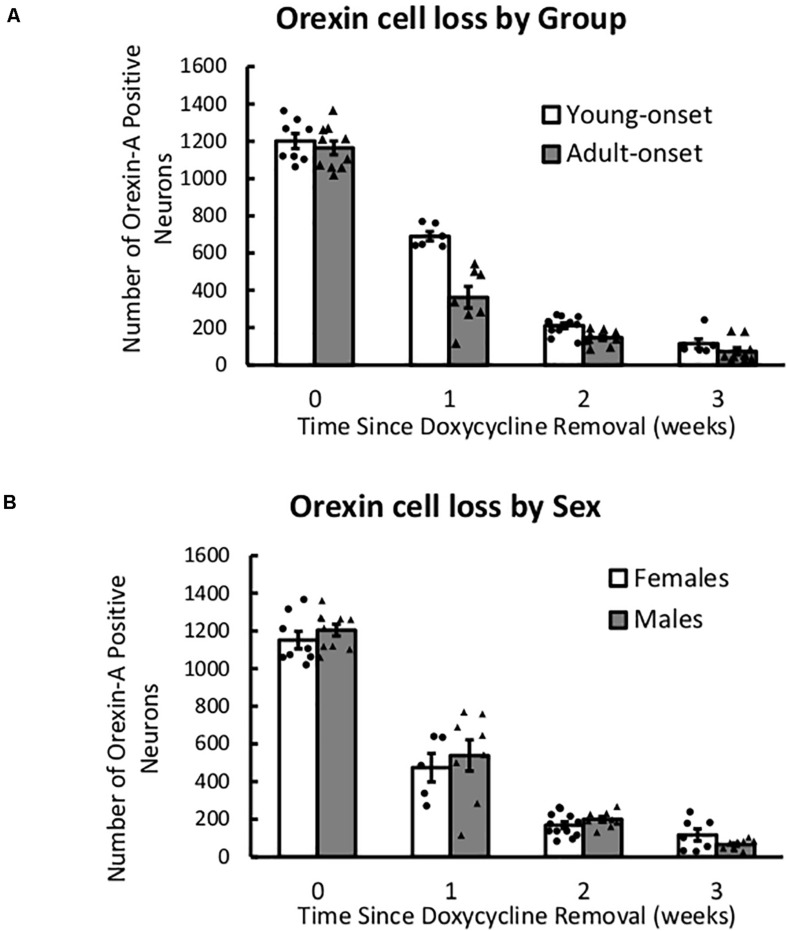
After DOX removal, orexin neurons are lost at similar rates in young- and adult-onset mice. **(A)** Average number of orexin-A positive neurons in young-onset and adult-onset mice over time since doxycycline removal. **(B)** Average number of orexin-A positive neurons in female and male mice over time since doxycycline removal.

After removal of DOX, the number of orexin neurons declined rapidly over 3 weeks, *F*(1) = 341.21, *p* < 2 × 10^16^, but this decline was unaffected by age of DOX removal or sex (Figure 7). There were no significant interaction effects (group × time, sex × time, or group × sex × time), indicating that there were no group differences in orexin count across time. Thus, any differences in behavior cannot be explained by differences in the rate of orexin neuron loss.

## Discussion

As cataplexy and sleepiness are often severe in children with narcolepsy, we investigated whether the symptoms of narcolepsy are worse when the orexin neurons are lost in young mice (onset 4 weeks) compared to adult mice (onset 14 weeks). We found that age of orexin neuron loss did not affect cataplexy severity, but female mice had more cataplexy than male mice and cataplexy duration tended to increase over time. Young-onset mice also tended to spend more time in REM sleep during the dark period than DOX+ controls. With both young- and adult-onset orexin neuron loss, wake bouts in the dark period were only about half the duration seen in controls, but there was no reduction in the total amount of wake over 24 h. Overall, this experiment did not support the hypothesis that young-onset orexin neuron loss would result in severe cataplexy, but it revealed an important sex difference in murine cataplexy and further characterized this important mouse model. These are the first experiments modeling young-onset narcolepsy in mice and a helpful step toward understanding the uniquely severe symptoms endured by children with narcolepsy.

### Effects of Age on Cataplexy

Young-onset and adult-onset mice spent a similar amount of time in cataplexy and had a similar number of cataplexy bouts. At 3 weeks after DOX removal, the adult-onset group spent about 2% of the dark period (∼15 min) in cataplexy which is similar to prior descriptions of adult *orexin-tTA; TetO DTA* mice (Tabuchi et al., 2014; Williams et al., 2019). However, Tabuchi et al. (2014) reported that at 13 weeks after DOX removal, adult-onset mice spent about 8% (∼60 min) of the dark period in cataplexy (Tabuchi et al., 2014). This amount is much higher than our group averages, although some individual mice in our experiments showed this large amount of spontaneous cataplexy. This discrepancy could be due to differences in the recording environment and equipment, inter-individual differences in scoring cataplexy, or background mouse strain (C57/BL6 mice vary slightly between Japan and the United States).

When narcolepsy begins in childhood, symptoms are often more severe than when narcolepsy begins in adults. Children tend to be sleepier than adults (Young et al., 1988; Pizza et al., 2013), and cataplexy tends to be more severe, persistent, and frequent (Serra et al., 2008; Plazzi et al., 2011; Antelmi et al., 2017; Maski et al., 2017). Beyond typical narcolepsy symptoms, childhood narcolepsy is also associated with precocious puberty and obesity, indicating widespread disruption to multiple systems (Kotagal et al., 1990, 2004; Plazzi et al., 2006; Vendrame et al., 2008; Poli et al., 2013; Ponziani et al., 2016). Indeed, children with narcolepsy are more likely to develop comorbidities affecting endocrine, metabolic, psychiatric, and nervous systems around diagnosis than age-matched healthy controls (Jennum et al., 2017). Our results suggest that this age-related difference may be specific to humans or may be a consequence of the autoimmune process hypothesized to kill the orexin neurons (Bonvalet et al., 2017), which is not modeled in these mice. For example, an especially aggressive autoimmune attack in children might kill the orexin neurons rapidly, leading to severe symptoms, and more disruption (Cogswell et al., 2019) whereas the neuron loss may occur more slowly in adults who develop narcolepsy. Alternatively, orexin neuron loss in adults may be less disruptive because of redundancies stabilizing different systems that have not yet developed in children.

Alternatively, some researchers maintain that narcolepsy progression is similar in children and adults but that children are typically studied closer to disease onset (Nevsimalova, 2009) or that only the most severe cases are diagnosed in children (Young et al., 1988). The first interpretation gains some preclinical support from the fact that *orexin-tTA; TetO DTA* mice removed from doxycycline at birth have far less cataplexy in adulthood than mice removed from doxycycline later (Tabuchi et al., 2014). Clinically, childhood cataplexy lessens over time and develops into a more typical, adult-like form (Plazzi et al., 2011; Pizza et al., 2013). The second hypothesis is possible as it usually takes years longer for children to be diagnosed with narcolepsy compared to adults (Guilleminault and Pelayo, 1998; Thorpy and Krieger, 2014; Maski et al., 2017), and milder cases may be overlooked. Either of these hypotheses could be supported by the lack of age effect shown here. However, because of the long diagnosis delay, most clinical studies are retrospective in nature, relying on patient and family recall of age and severity of symptoms at onset. This major limitation to much of the clinical data further emphasizes the importance of longitudinal studies in children with narcolepsy and studying age of narcolepsy onset in animal models, which provide better control than clinical research.

### Sex Differences in Cataplexy

We found that female mice have more cataplexy than male mice, yet researchers debate whether narcolepsy prevalence differs between men and women. Some early epidemiological studies indicate that narcolepsy is more common in men than in women (Silber et al., 2002; Ohayon et al., 2005; Longstreth et al., 2009). However, studies that separated narcolepsy type 1 (with cataplexy) from narcolepsy type 2 (without cataplexy) found a much smaller effect (Silber et al., 2002), no sex difference in NT1 (Ohayon et al., 2002; Heier et al., 2009; Luca et al., 2013; Khatami et al., 2016), or a higher incidence in women (Longstreth et al., 2009). Given the significant diagnosis delay and frequent misdiagnoses of patients with narcolepsy (Guilleminault and Pelayo, 1998; Kryger et al., 2002; Macleod et al., 2005; Thorpy and Krieger, 2014; Maski et al., 2017), it is likely that older reports underestimated the prevalence of narcolepsy, especially in women. Importantly, considering the typical finding of an increased prevalence in men, the diagnostic delay is typically longer in women than in men (Luca et al., 2013), and women are less likely to be assessed in sleep laboratories, resulting in less access to polysomnography and likely underdiagnosis of sleep disorders (Auer et al., 2018). Overall, it seems unlikely that there is a significant sex difference in the prevalence of narcolepsy type 1 in humans.

Most studies do not assess gender differences in cataplexy severity. Cataplexy may be more common in women than in men (Ohayon et al., 2002), but there seems to be no difference in severity between men and women (Luca et al., 2013). One study found that a larger proportion of men with “high frequency” cataplexy (more than one bout a month), but the number of patients was relatively low (44) and there were few women in the study (16) (Mattarozzi et al., 2008). Future clinical studies should examine gender as a possible factor contributing to cataplexy severity.

While we suspect that the sex difference in cataplexy reported here is unlikely to parallel human narcolepsy, it may arise from thermoregulatory influences. Cataplexy is a REM sleep-like state, and REM sleep and cataplexy are likely regulated similarly. Warmer ambient temperatures increase REM sleep, and cooler temperatures decrease REM sleep in rodents (Schmidek et al., 1972; Szymusiak and Satinoff, 1981; Kumar et al., 2009; Komagata et al., 2019). Female mice prefer warmer temperatures than males, suggesting that the thermoneutral zone for females may be slightly warmer compared to males (Gaskill et al., 2009; Kaikaew et al., 2017). While cooler temperatures inhibit REM sleep, they may be more permissive to cataplexy. In the current experiment, mice were housed at 22°C, and this cool temperature may have inhibited REM sleep more in females than in males, yet persistent REM sleep pressure may have resulted in more cataplexy in females.

In addition, the estrus cycle can influence orexin levels and sleep/wake behavior. Hypothalamic orexin levels are higher during proestrus than in other stages of the estrus cycle in rats (Porkka-Heiskanen et al., 2004); however, we are not aware of any studies examining cataplexy across the estrus cycle. While the effects of the estrus cycle on sleep/wake architecture in female mice are modest and strain-specific, REM sleep is significantly reduced in the dark period during proestrus in female Sprague-Dawley rats and C57/BL6 mice (Fang and Fishbein, 1996; Koehl et al., 2003). Interesting, male rats spend more time in REM sleep than female rats overall, a finding that our results support, although a previous study in mice did not find this difference (Fang and Fishbein, 1996; Paul et al., 2006). Despite the likelihood that the sex difference shown here is a species-specific effect, this aspect of murine cataplexy has implications for future research. Cataplexy is an uncommon state, so including females may help increase the overall number of cataplexy bouts seen across groups, in addition to better modeling the patient population.

### Effects of Orexin Neuron Loss on REM Sleep

Young-onset mice tend to spend more time in REM sleep during the dark period than DOX+ controls, although oddly, adult-onset mice did not show the same pattern. An increase in dark period REM sleep was expected as the orexin neurons are thought to normally suppress REM sleep during the dark period (Kantor et al., 2009). Kantor et al. (2009) demonstrated this disinhibition of REM sleep during the subjective night in mice housed in constant darkness, and this effect has also been shown in orexin-ataxin mice and orexin knockout mice maintained on a 12:12 light:dark cycle (Hara et al., 2001; Kantor et al., 2009; Roman et al., 2018; Chowdhury et al., 2019).

Interestingly, this increase in REM sleep during the subjective dark period was originally shown in orexin-ataxin-3 mice but not orexin knockout mice, which was thought to indicate an effect of the loss of orexin neurons as opposed to the loss of the orexin peptides *per se* (Kantor et al., 2009). Our results are consistent with this hypothesis, but it is also possible that this REM sleep effect is seen soon after the loss of the orexin neurons but before some compensation occurs. This hypothesis is supported by the fact that *orexin-tTA; TetO DTA* mice that are removed from DOX in adulthood have more REM sleep in the dark period 3 weeks after DOX−, but not 4–13 weeks after DOX removal, and *orexin-tTA; TetO DTA* mice removed from DOX at birth show no increase in dark period REM sleep (Tabuchi et al., 2014). However, further research will be necessary to parse out the acute and chronic effects of orexin neuron loss vs. orexin peptide loss on REM sleep in the dark period.

### Limitations

A few limitations in these experiments warrant discussion. We removed DOX from the chow of 4-week-old mice as they are roughly the same developmental stage as 11–12 years old children (Flurkey et al., 2007), but it is difficult to accurately align human ages onto mice. It is possible that orexin neuron loss prior to 4 weeks of age would produce more severe cataplexy. It is also important to consider that all cataplexy reported here is spontaneous, but the addition of palatable food (Froot Loops or chocolate) or a running wheel can dramatically increase cataplexy in orexin knockout mice (Espana et al., 2007; Clark et al., 2009; Burgess et al., 2013; Oishi et al., 2013; Mahoney et al., 2017), and young-onset mice may be more vulnerable to reward-elicited cataplexy. We did not control for estrus cycle in this experiment, which likely accounts for some of the variability in cataplexy and REM sleep in female mice; however, our data from females are only slightly more variable than males. Thus, while it is best practice to control for estrus cycle, we do not believe that this should be a barrier to including females in experiments.

## Conclusion

In contrast to our hypothesis, we did not find more cataplexy in mice with orexin neuron loss at a young age. Instead, we found that female mice have more cataplexy overall than male mice, which is likely a species-specific effect that can aid narcolepsy research. Still, the hypothesis is open, and future research could determine whether DOX removal at birth or at weaning results in severe cataplexy in mice; examine whether age of orexin neuron loss affects reward-elicited cataplexy; characterize cataplexy in female mice as a function of estrus cycle; further parse out the acute and chronic effects of orexin neuron loss vs. orexin peptide loss on dark period REM sleep; or investigate compensatory mechanisms that develop in the weeks after orexin neuron loss. Children are uniquely burdened by narcolepsy symptoms, and research dedicated to this population is necessary to address this problem.

## Data Availability Statement

The raw data supporting the conclusions of this article will be made available by the authors, without undue reservation.

## Ethics Statement

The animal study was reviewed and approved by the Institutional Animal Care and Use Committee of Beth Israel Deaconess Medical Center and Harvard Medical School.

## Author Contributions

AC and TS conceived of and designed all experiments. AY provided mice for the experiments. AC performed surgeries, recordings, perfusions, sleep stage scoring, and data analysis and drafted the manuscript. AJ performed mouse husbandry/weaning, perfusions, brain sectioning, immunostaining, imaging, image analysis, and orexin neuron counting and drafted the relevant methods section. TS, AY, and AJ made significant revisions to the manuscript. All authors contributed to the article and approved the submitted version.

## Conflict of Interest

The authors declare that the research was conducted in the absence of any commercial or financial relationships that could be construed as a potential conflict of interest.

## Supplementary Material

Supplementary Videos Example videos of cataplexy bouts in SleepSign. EEG (top) and EMG (bottom) signals are presented for two representative cataplexy bouts. The three panels in the bottom left corner (from top to bottom) show: Delta Power, EMG integral, and Theta Ratio. Epochs are labeled “W” for wake or “C” for cataplexy. Asterisks indicate that the mouse transitioned between states within the 10-s epoch. In these cases, the epoch was scored as the state lasting for at least 50% of the epoch. In both mice, the EEG signal during cataplexy resembles REM sleep, the mouse is immobile for the duration of the bout, and theta activity dominates the EEG activity. Hypersynchronous theta activity is visible as brief bursts of larger amplitude EEG signal. **Video 1** Example cataplexy bout in a young-onset mouse (age 17 weeks). The cataplexy bout begins about 8 s into epoch 756 (blue numbers beneath EMG signal). The bout ends 6 s into epoch 771. **Video 2** Example cataplexy bout in an adult-onset mouse (age 17 weeks). The cataplexy bout begins 4 s into epoch 1262 (blue numbers beneath EMG signal). The bout ends 5 s into epoch 1267.

## Abbreviations

DOX: doxycycline
DOX −: mice removed from doxycycline
DOX+: mice maintained on doxycycline.
